# Diet in chronic kidney disease in a Mediterranean African country

**DOI:** 10.1186/s12882-017-0448-2

**Published:** 2017-01-23

**Authors:** Khawla Kammoun, Hanen Chaker, Hichem Mahfoudh, Nouha Makhlouf, Faical Jarraya, Jamil Hachicha

**Affiliations:** 1 Neprology Department Hedi Chaker Hospital, Sfax, Tunisia; 2 Renal Pathology Unit, UR 12 ES 14 Medecin, University Sfax Tunisia, Sfax, Tunisia

**Keywords:** Chronic renal failure, Diet, Low protein diet, Phosphorus, Potassium, Sodium

## Abstract

**Background:**

Mediterranean diet is characterized by low to moderate consumption of animal protein and high consumption of fruits, vegetables, bread, beans, nuts, seeds and other cereals. It has been associated with reduced risk of cardiovascular disease. However, it is not suitable for chronic kidney disease because of high potassium intake.

**Discussion:**

Tunisia is an emerging Mediterranean country with limited resources, a high prevalence of chronic hemodialysis treatment and high dialysis expenditures. In order to limit dialysis cost, primary and secondary prevention of chronic renal disease are of paramount importance. In addition to drugs, secondary prevention includes diet measures (e.g. salt diet, protein diet). The aims of diet practice in chronic kidney disease are to slow chronic renal failure progression and to prevent its complications like hyperphosphatemia and hyperkaliemiae. A few decades ago, a Tunisian diet was exclusively Mediterranean, and protein consumption was not excessive. However, today, protein consumption is more comparable to western countries. Salt consumption is also excessive. Some Tunisian diets still include food with high potassium intake, which are not suitable for patients with chronic kidney disease. Therefore, the role of the dietician is extremely important to help calculate and create a dietary regimen tailored to each of our patients.

**Summary:**

Advice about diets should be adapted to both the patient and population habits to improve adherence rate. As such, the purpose of this article is to provide our own experience regarding medical nutrition therapy in patients with chronic kidney disease in Tunisia, with some changes in food habits. Prevention is far better than treatment. In this perspective, dietary measures must be at the core of our intervention.

## Background

Tunisia is an emerging country with limited resources (https://en.wikipedia.org/wiki/Emerging_markets#cite_note-2, https://www.theisn.org/images/Membership/Eligible_Countries_for_Joint_Membership_Jan_2016.pdf, https://datahelpdesk.worldbank.org/knowledgebase/articles/378834-how-does-the-world-b). Chronic hemodialysis treatment begun in Tunisia in 1963 with a very strict selection. Only young people with social insurance, and without severe co morbidities (like neoplasia or severe heart disease) were treated with dialysis. End stage renal disease incidence rose from 81.6 per million people (pmp) to 137 in 2007 [1, 2]. This sharp increase could be linked to a political decision made in 1991 to treat all patients, regardless of their social insurance and comorbidities, but without significant increased number of renal transplantation which was at 14 pmp in 2007. Prevalence of renal replacement therapy (RRT) in December 2007 was 713 pmp [2]. Sfax is a southern Tunisian city, with one million population. According to the last regional registry data in 2014 RRT prevalence was 771 patients.

The main causes of end stage chronic kidney disease are unknown nephropathy [3]. Increase in the prevalence of ESRD treated with dialysis has led to an increase in costs of dialysis treatment, and dialysis expenditures represented 4.5% of Tunisian Health budget in 2000 [1]. Primary and secondary CKD prevention is therefore essential to our country which has limited resources. Primary prevention aims to decrease CKD incidence. Secondary prevention aims to slow renal function decline. They include not only therapeutic measurement such as control of hypertension, diabetes, but also lifestyle and dietary measures such as restricting sodium and protein intake.

Protein intake is still low in several Sub-Saharan African countries [4]. In Tunisia, protein intake appears to be adequate and similar to that observed in migrant Tunisian people living in France and local born French people [5]. In our center, we conducted a dietary intake evaluation prospective study of 100 consecutive CKD patients. Mean protein intake was 1.48 ± 0,4 g/kg/d (0.73-3 g/kg/d). We found that only 6 patients had a protein intake < 1 g/kg/d and 4 patients had a protein intake exceeding 2 g/kg/d [data not published].

Since the Brenner theories of increased workload on the remnant nephrons, and its prevention by decreasing protein intake [6], use of low protein diet for patients with renal damage in patients with CKD is a subject of controversy between supporters and adversaries [7, 8]. Complications of renal failure such inflammation and acidosis lead to hypercatabolism by activating enzymes that breakdown proteins. However, hypercatabolism is not stopped by protein intake increase. An increase in protein intake will worsen acidosis and accumulation of potential uremic toxins and development of uremic symptoms without increasing muscle mass [9, 10]. It has been shown that protein intake reduction in patients with moderate chronic kidney failure reduces blood chemistry abnormalities like acidosis, phosphorus and urea [11].

The main objective of any diet practice in patients with CKD is to slow chronic renal failure progression without inducing malnutrition. So before any prescription of protein diet, a nutrition assessment status should be performed. Regular nutritional assessment should be conducted every 2-3 months for non-dialysed out-patients with glomerular filtration rate (GFR) <20 ml/mn is recommended [12]. Screening for malnutrition should be more frequent when there is a malnutrition risk event. There are many methods for screening malnutrition, but the method selected should be simple, reproducible and not expensive [12].

The nutritional status assessment should be based on a group of clinicobiological parameters, which we employ in our center. These parameters should be inexpensive, reproducible and accessible in routine practice.

### Antrophometric parameters

Measurements of height, actual body weight and its comparison to an anterior body weight or to an ideal weight, and body mass index (BMI) calculation are easy simple and inexpensive methods to assess nutritional status. In our center, these evaluations are conducted at each visit by a nephrologist. However, one limitation of this method is body sensitivity to hydration modification status [12]. Measurement of four skinfold thicknesses (biceps, triceps, subscapular, and suprailiac) is a validated method to estimate fat mass and lean body mass. We carried out a cross sectional study in hemodialysed patients, which involved comparisons between routine anthropometric measurements and skinfold thicknesses measurements, and found a significant correlation between BMI and skin-fold measurements. However, unlike BMI, these techniques need an experienced technician and is time consuming. As such, it cannot be used in routine practice [13, 14].

### Laboratory assessment

Serum proteins that are considered as nutritional markers are albumin, prealbumin tranferrin, and retinol binding protein, but no serum protein has been found to be specific for malnutrition. No biological marker has a high sensitivity, or specificity for screening nutritional state, and the protein sensitivity for nutrition assessment depends on the duration of their individual half-lives. Their concentration could also change in other physiologic and pathological situations [13, 15–20]. In our center, serum albumin is used to evaluate nutritional status when malnutrition is clinically suspected. In Tunisia, assessment of serum albumin levels is recommended every 6 months in dialyzed patients. There is no national recommendation in non-dialyzed CKD patients.

### Index Score - subjective global assessment (SGA)

Malnutrition is one of the main important risk factors of inflammation and atherosclerosis in chronic renal failure. Several studies have shown that physical examination scores like subjective global assessment (SGA), malnutrition inflammation score (MIS) and nutritional risk screening (NRS) detect prognostic of malnutrition more reliably than laboratory parameters alone [21]. It can recognize various degrees of malnutrition that may remain undetected by a single laboratory parameter [22]. SGA can be used to determine malnutrition outcome, and as such is more commonly used [23].

The SGA score is correlated to anthropometric parameters such as mild arm circumference and skinfold measurement. It is a simple method using clinical and anthropometric parameters that are easy to evaluate [24]. In a cross-sectional study in hemodialyzed patients, we demonstrated that SGA has a high specificity (86%) and a good positive predictive value compared to estimation of fat and lean mass using four skinfold thicknesses (biceps, triceps, subscapular, and suprailiac) and arm circumference [14]. SGA is recommended by the European Best Practice Guidelines on Nutrition and the national kidney foundation as a valid method to identify patients at risk of malnutrition [25, 26].

In our practice, when a patient is identified as suffering from malnutrition, serum albumin levels are checked, and the patient is referred to the dietician who evaluates their nutritional status using SGA, and will prescribe dietary advice according to their nutritional status, CKD stage and co morbidities.

## Protein diet

When prescribing protein intake, normal, low and high protein intake should be defined. However, normal protein intake definition is not clear. It should be a diet containing a minimum requirement to avoid deficit but also a not surpassing an ideal intake. The recommended protein intake is the daily intake level sufficient to meet the nutriment requirement of 97-98% of healthy people (https://ods.od.nih.gov/Health_Information/Dietary_Reference_Intakes.aspx) [27]. The recommended daily protein intake of healthy individuals is 0.83 g/kg/day [28].

An intake of 0.66 g/kg/day is accepted as average intake. The safe level of intake is equivalent to 0.83 of high quality protein [29].

In most studies restrictive protein and phosphorus diet delay glomerular filtration decline. But low protein diet < 0.6 g/kg is not recommended because their benefit is little and they increase malnutrition risk [30].

In our center we do not prescribe a restrictive diet below 0.6 g/day. A more restrictive diet needs a very strict monitoring of nutrition state and it has been shown not to have a significant benefit in many studies [31]. Moreover we have only one dietitian for 120 regular dialyzed patients and 2000 hospitalizations and 2000 consultations per year. In practice, after the second or the third nephrology consultation, patients with chronic renal failure, are referred to dietician for diet advice. Our dietician performs a nutritional status assessment using a anthropometric parameters (weight, height, and body mass index a dietary) and SGA. Following this, the patient is provided with diet advice and meal menu examples based on their Tunisian habits and lifestyle (Tables 1, 2 and 3). In general practice, for patients with middle and high socioeconomic status, the dietician will advise patients to eat animal protein (such as meat and fish) for either lunch, or dinner (Tables 1, 2 and 3).

**Table 1:** Example 1: for a 60 kg women: 1550 Kcal, 53% carbohydrates, 12% protides and 35% lipids 2400 mg K+, 849 Ph

	Menu's examples
breakfast	Milk 200 ml
	bread: 50 g
	olive oil 1 ts^a^
	1 fruit
lunch	Vegetables^b^: 200g
	meat or fish 80 g
	rice or paste 300 g or white bread:120g
	olive oil 1 cs
dinner	Vegetables^b^: 200g
	meat: 0
	rice or paste 200 g or white bread:80 g
	olive oil 1 ts^a^
	1 fruit^c^

^a^ts: tablespoon; ^b^double cooked; ^c^: 1 fruit= 1apple=1 peach =3 apricot=50g grenade

**Table 2:** Example 2: for a 60 kg women: 1550 Kcal, 53% carbohydrates, 12% protides and 35% lipids 2400 mg K+, 849 Ph

	Menu's examples
Breakfast	Milk 200 ml
	bread: 50 g
	olive oil 1 ts^a^
	1 fruit, 1 yogurt
lunch	Vegetables^b^: 200g
	1 egg
	rice or paste 300 g or white bread:120g
	olive oil 1 cs
dinner	Vegetables^b^: 200g
	meat : 0
	rice or paste 200 g or white bread:80 g
	olive oil 1 ts^a^
	1 fruit^c^, 1 yogurt

^a^ts: tablespoon; ^b^double cooked; ^c^: 1 fruit= 1apple=1 peach =3 apricot=50g grenade

Vegetables example 1: double cooked Chard, Spinach

Vegetables example 2: Tunisian salad: finely chopped Cucumber + tomato+onion+Pepper

**Table 3:** Example 3: for a 70 kg men: 2000 Kcal, 49% carbohydrates, 12% proteins and 39% lipids 2196 mg K+, 998 mg Ph

	Menu's examples
breakfast	Milk 200 ml
	bread: 80g
	olive oil 1 cs
	1 yaourt
lunch	Vegetables^b^: 200g without potatoes
	meat 100 g
	rice or paste ex 300 g
	olive oil 1 ts
dinner	Vegetables^b^: 200g without potatoes
	meat: 0
	rice or paste 250 g
	olive oil 1 ts^a^

^a^ts: tablespoon; ^b^double cooked; 1 fruit= 1apple=1 peach =3 apricot=50g grenade

## Phosphorus

Hyperphosphataemia is an ineluctable consequence of chronic renal failure with serious complications at short, middle and long term outcomes. Hence, it is a veritable challenge of the nephrologist in the follow up of his patients. Some authors have emphasized the importance of early and effective control of phosphate load before hyperphosphataemia develops to prevent the increase in PTH and FGF23 and maintain near-normal phosphorus levels for longer as CKD progresses [32].

Dietary measure are crucial in hyperphosphatemia, even if drugs are needed.

In this approach, patient, nephrologist and dietician together form an equilateral triangle with the apex as the patient. Collaboration with dietician improves dietary compliance. Restriction of food containing phosphorus is not prescribed until hyperphosphatemia is present [28, 32]. It has been shown that dairy intake significantly increased the serum phosphorus concentration at different DFG levels [29, 32, 33], so they must be consumed with moderation. However, some aliments are prohibited, because of the high content of phosphorus, potassium and sodium like soft drinks, and canned foods. Some other foods have high phosphorus and sodium content (such as processed and melted cheese, delicatessen and aliments with phosphorus additives) should be also avoided.

Our experience of diet management is based on a preliminary upkeep with the nephrologist who explains the risk of hyperphosphataemia to the patient, and that the main treatment is the reduction of aliments rich in phosphorus (e.g. dairy, legumes, meat, complete cereals, dried fruit, and soda drinks). The patient is then referred to department dietician. As mentioned previously, our department has only one dietician for 120 dialyzed patients and 2000 hospitalizations and 2000 consultations per year. For external consultation the dietician provides weekly consultations for CKD and hypertensive patients The dietitian begins with a food consumption survey in order to detect the patient’s excess. (The phosphorus intake should be 800 -1000 mg/d). They explain to the patient that some food products must be avoided because of their high phosphorus and sodium content, such as octopus and particularly cuttlefish, which is consumed excessively in our local area, as many coastal towns in this region have high fish consumption. Some food products should be taken in moderation, such as dairy, legumes, meat, complete cereals and dried fruit (Table 4).

**Table 4:** Phosphorus concentrations in examples of food regularly taken in our Habits

	Mg/100 g (Ph)	G/100g(proteins)
Veal, Lamb, beef	115-202	24,6-30
poultry (Chicken, Turkey)	170-200	23-28
barley semolina	296 mg	10,5
Chickpea	139	8,9
Sorghum	287	11,3
octopus, squid, cuttlefish	127	16
Whiting	196	18,7
sea bass	256	22,4
sea mullet	244	24,8
grilled sardine	320	30
tuna	220	23,7-29,9
Canned Sardine	490	23
Canned Tuna	267	25,6

It is common in our region that men and elderly people don’t cook for themselves when they do not live alone. If the patient confirms that they will not be preparing their own food, our dietician will ensure that they discuss diet changes and the new recipes with the person who will be preparing the patients’ food, again, to help improve adherence to the new diet. These recipes should be adapted to the patient's socio-demographic characteristics. For example, the rural patient does not have the same meal habits as those living in towns or cities. In fact, in our geographic region, eating meals prepared outside of the home is more common in in cities where there is greater animal product consumption, while vegetable staples and full grain are generally consumed more in rural regions. It is therefore very important that the dietician works closely with the patient and their family to obtain a clear understanding of their meal habits so that they can create meal examples that cater to the patient’s habits and lifestyle. If the patient is provided with a diet plan that has been tailored to their lifestyle, then the patient is more likely to adhere to these changes.

### Iron

Iron is one of the minerals that should be given special attention [17]. The general population requires 10-15 mg/day of iron, but patients with renal failure and ferritin levels < 100 mg/dL should receive supplements of at least 60 mg/day of ferrous sulfate. Concomitant intake of iron with a citrus juice, promoting iron absorption together with vitamin C, may be suggested. The choice of a specific iron drug can be based on price, since the published literature contains few comparative studies which establish the superiority of a particular agent. Generic ferrous sulfate, 325 mg orally 3 times daily between meals, supplies 195 mg of elemental iron and may be an appropriate choice. If gastrointestinal side effects develop with oral iron supplements, the dose should be decreased. Clinicians should not react to gastric distress by instructing patients to take the supplements with meals, which will result in greatly limited iron absorption.

### Vitamin D

Vitamin D is commonly deficient in chronic renal failure. This finding could be explained by a combination of factors, including poor nutrition, gastrointestinal disorders, or a lack of vitamin D synthesis because of little exposure to sun light [34]. Severe deficiency causes hypocalcaemia and induces hyperparathyroidism. Vitamin D is found in some foods, and from dermal synthesis from sun exposure. As such, supplementation is commonly needed, however the quota from animal proteins may protect from severe deficits [35]. It is commonly known that animal proteins are rich of vitamin D (for example, tuna, sardines, eggs, and veal liver). Nevertheless, vegetables aren’t a common source of vitamin D, with the exception of mushrooms, which isn’t currently consumed in our region.

The inconvenience of these aliments reside on their high phosphorus content (for example eggs), or uric acid (tuna, sardines).

Therefore, the role of dietician and their guidance is paramount when selecting the patient's food choices in order to consume the adequate quantity of animal proteins which will provide the body with the essential amino acids for tissue repair, and hormone synthesis without inducing excess consumption of phosphorus.

The dietician must calculate and integrate the patient’s need for calories, vitamins, amino acids and hormones, and the result is a recipe that has been adapted specifically to the eating patient's habits.

This is a very demanding role for the dietician, and it is therefore important that the national authorities are made more aware about the need to increase the number of dieticians in public hospitals. It is an ineluctable issue in the future, especially with the outbreak of the obesity, diabetes, hypertension and chronic renal failure. A cross sectional national study, conducted in 2012, showed that hypertension, obesity, diabetes and metabolic syndrome among 35-70 years old people was 30.6%, 27.3%, 9.1% and 30% more frequent in women living in urban regions, respectively [36] (http://www.letemps.com.tn/article/88107/sant%C3%A9-lutte-contre-l%E2%80%99hypertension-le-diab%C3%A8te-l%E2%80%99ob%C3%A9sit%C3%A9-et-les-maladies-cardiaques-pour). Prevention has always been far away better than treatment, and with this in mind, the diet therefore must be in the core of our intervention.

### Sodium

There is a close link between sodium intake and progression of renal disease, but with an increased consumption in dietary salt, people are becoming more accustomed to having high salt content in their food.

Dietary salt sources in Tunisian are different to those in Europe for example. Interestingly, table salt, pre-prepared or processed food is not the major source of dietary salt – salt added to food only represents 0.25% of salt consumption in the Tunisian diet. In our population the primary source comes from bread, which is not only rich in salt, but it forms the basis of the daily diet in Tunisia, and represents 30% of salt consumption. Other traditional sources of salted food, such as dried meat, are not as common today.

In December 2014, a press communication from the Tunisian Ministry of Health indicated that daily mean consumption of salt in Tunisia is 10 g/person. With an aim to reduce salt consumption to 5 g/day, new strategies were proposed by the Ministry of Heath with a focus on reducing salt use in bread in a pilot study in the city of Bizerte (http://www.letemps.com.tn/article/88107/sant%C3%A9-lutte-contre-l%E2%80%99hypertension-le-diab%C3%A8te-l%E2%80%99ob%C3%A9sit%C3%A9-et-les-maladies-cardiaques-pour). In addition, a cost effectiveness analysis of salt reduction policies to reduce coronary heart disease was conducted in Mediterranean countries, including Tunisia. It concluded that a comprehensive strategy of health education and food industry actions to label and reduce salt content would save both money and lives [37, 38].

Whilst we are waiting for the results from the pilot study, we continue to educate our patients about dietary salt. When salt restriction is recommended, the patient is requested to limit bread and preserved food use, especially canned tomatoes, peppers, tuna and sardines, which are widely used in Tunisian food. This can be a difficult task in a poor country, where bread is the cheapest food in Tunisia, and tuna and sardines are easy to come by in coastal regions. In a CKD patient’s clinical follow-up, salt consumption is evaluated by considering the total amount of 24 h urine sodium. This checkup is not frequently used by nephrologists; however, advice regarding salt restriction is commonly given to patients.

## Potassium (K+)

In Tunisia, a Mediterranean diet is predominant, and as such, a high amount of vegetables and fruits are consumed which leads to a high potassium intake. If such a diet is recommended for cardiovascular protection, in the very high cardiovascular risk CKD patient, it becomes problematic. When serum potassium levels are at high normal range, we recommend patients to avoid dates, the highest potassium content product, largely consumed as a fruit in Tunisia. For the others, we recommend a diet with a reduced intake of specific high potassium foods, such as bananas, dried fruits, almonds, vegetables and chocolate (Table 5). Our advice is not to soak vegetables in water, which is not effective at reducing potassium levels, but instead double cook them (for example, boil, rinse and boil again). This process leaches more potassium from the vegetables than normal cooking methods [37].

**Table 5:** Potassium concentrations in examples of food regularly taken in our habits

	(K+) Mg/100g
datte	2600 mg
barley semolina	309
Chickpea	168
dry bean	460
Lens	276
Cucumber	150
Lettuce	234
tomato	226
onion	170
Cabbage	293
Pepper	170
Chard	378
Parsley	800
Spinach	529
Fennel	430
Sorghum	351

For raw vegetables used in salads, it is advised to use original Tunisian salad containing tomatoes, cucumber, onion and peppers. Other vegetables, such as cabbage, should be avoided in salads.

## Conclusion

Our main objective of diet prescription in CKD is to slow chronic renal failure progression and to prevent its metabolic complications such as hyperphosphatemia and hyperkaliemia. Dietary restriction covers three principal components: potassium, phosphorus and sodium.

Potassium restriction needs to avoid certain fruit and vegetables. Phosphorus restriction that needs protein intake restriction should be carefully prescribed to avoid malnutrition. Dietary salt should also be reduced.

The role of the dietician is therefore pivotal in helping to create such a diet. For each patient, the dietician must determine their nutritional state, and take into consideration the patient’s individual lifestyle habits. This also involves providing guidance and support to the patient, and their family if required. Nutritional advice should be general but it is important to also adapt the diet to each patient’s eating habits to improve adherence rates.

The diet itself must offer an adequate quantity of animal protein, which will provide the body with the essential amino acids for tissue repair, hormone synthesis without inducing phosphorus, and balance the level of sodium or potassium consumption. It’s a veritable calculation which must also integrate the patient’s requirement for calories, vitamins, amino acids and hormones.

Given the low number of dieticians working in our hospital settings, local authorities need to be made more aware of crucial role played by dieticians. Prevention is far better than treatment. In this perspective, dietary measures must be at the core of our intervention.

## Abbreviations

ABW: Actual body weight
BMI: Body mass index
CKD: Chronic kidney disease
GFR: Glomerular filtration rate
IBW: Ideal body weight
K+: Potassium
MIS: Malnutrition inflammation score
NRS: Nutritional risk screening
Ph: Phosphorus
SGA: Subjective global assessment
